# Maize disease detection based on spectral recovery from RGB images

**DOI:** 10.3389/fpls.2022.1056842

**Published:** 2022-12-21

**Authors:** Jun Fu, Jindai Liu, Rongqiang Zhao, Zhi Chen, Yongliang Qiao, Dan Li

**Affiliations:** ^1^ College of Biological and Agricultural Engineering, Jilin University, Changchun, China; ^2^ Key Laboratory of Efficient Sowing and Harvesting Equipment, Ministry of Agriculture and Rural Affairs, Jilin University, Changchun, China; ^3^ Key Laboratory of Bionic Engineering, Ministry of Education, Jilin University, Changchun, China; ^4^ Department of Science and Technology Development, Chinese Academy of Agricultural Mechanization Sciences, Beijing, China; ^5^ Australian Centre for Field Robotics (ACFR), Faculty of Engineering, The University of Sydney, Sydney, NSW, Australia; ^6^ College of Astronautics, Nanjing University of Aeronautics and Astronautics, Nanjing, China

**Keywords:** maize, pest disease detection, spectral recovery, hyperspectral images (HSIs), convolutional neural network (CNN)

## Abstract

Maize is susceptible to infect pest disease, and early disease detection is key to preventing the reduction of maize yields. The raw data used for plant disease detection are commonly RGB images and hyperspectral images (HSI). RGB images can be acquired rapidly and low-costly, but the detection accuracy is not satisfactory. On the contrary, using HSIs tends to obtain higher detection accuracy, but HSIs are difficult and high-cost to obtain in field. To overcome this contradiction, we have proposed the maize spectral recovery disease detection framework which includes two parts: the maize spectral recovery network based on the advanced hyperspectral recovery convolutional neural network (HSCNN+) and the maize disease detection network based on the convolutional neural network (CNN). Taking raw RGB data as input of the framework, the output reconstructed HSIs are used as input of disease detection network to achieve disease detection task. As a result, the detection accuracy obtained by using the low-cost raw RGB data almost as same as that obtained by using HSIs directly. The HSCNN+ is found to be fit to our spectral recovery model and the reconstruction fidelity was satisfactory. Experimental results demonstrate that the reconstructed HSIs efficiently improve detection accuracy compared with raw RGB image in tested scenarios, especially in complex environment scenario, for which the detection accuracy increases by 6.14%. The proposed framework has the advantages of fast, low cost and high detection precision. Moreover, the framework offers the possibility of real-time and precise field disease detection and can be applied in agricultural robots.

## 1 Introduction

Maize is one of the most vital food and industrial crops for human beings and is the most essential cereal crop across the globe after rice and wheat (Haque et al. (2022)). In addition to its edible value, maize also serves as the raw material for industrial products and animal fodder (Demetrescu et al., 2016; Samarappuli and Berti, 2018; He et al., 2018). However, maize is susceptible to various pest diseases (Mboya, 2013), and the loss of maize yield induced by pest disease has increased sharply. Early detection is an important way to stop the spread of pest diseases, but expert identification is time consuming and high cost. Therefore, the computer vision and machine learning technique has attracted numerous attention for detecting infected plants (Chen et al., 2021; Feng et al., 2020; Feng et al., 2021).

The raw data commonly used for disease detection is RGB images which are generally acquired by digital camera. Several disease detection models which combine RGB images with machine learning were proposed in recent years. Zhang et al. (2021) proposed a convolutional neural network (CNN) model optimized by a multi-activation function module in order to detect maize diseases including maculopathy, rust and blight. Wu (2021) introduced a two-channel CNN which constructed based on VGG and ResNet for maize leaf diseased detection and achieved a better performance than the single AlexNet model. A CNN model based on transformer and self-attention was implemented to automatically identify maize leaf diseases in a complex background (Qian et al. (2022)). Due to the high efficiency and low cost in RGB data acquisition, RGB image is the first choice for training deep learning model. However, most of the current models trained by RGB data are image-wise classification of plant diseases (Karthik et al. (2020); Wang et al. (2021); Syed-Ab-Rahman et al. (2022)). In the application in field, precise positioning of the diseased area is needed. Therefore, pixel-wise detection plays an important part in plant disease detection, but RGB image only has 3 channels in spectral domain and barely capable of locating diseased area accurately on account of the deficiency of spectral information.

Hyperspectral image (HSI), regarded as high-dimensional data can provide tremendous information on spectral domains. HSI, not like RGB image which only has three spectral bands, has multiple bands could be used for extracting disease characteristics, so it is an ideal candidate for pixel-wise disease detection (Nagasubramanian et al. (2019); Zhang et al. (2020); Feng et al. (2021)). Nguyen et al. (2021) extracted disease features from HSI data cube to detect grapevine vein-clearing virus and accomplished pixel-wise classification by using random forest classifier. By selecting features from shortwave infrared HSIs of peanuts, Qiao et al. (2017) concentrated spectral information into a subspace where the healthy peanuts and fungi-contaminated peanuts can be separated easily. Although HSI could not only provide amounts of spectral information but also locate the infected area effectively, the drawbacks of HSI are also observed. Normally, owing to the measurements of hyperspectral camera are performed based on the line scanner, the time to obtain HSI data is much longer than get RGB image by digital camera (Behmann et al. (2018)). Hence, it is hard to complete the disease detection fast and efficiently in the application of field detection. Moreover, the cost of hyperspectral imaging system is much higher than digital camera, so it is difficult to spread the use of it.

Above all, using neither RGB images nor HSIs could combine the advantages of detection accuracy, detection speed, data acquirement, and low cost. Ideally, it would be great if we could acquire HSI through a digital RGB camera. In this way, we can keep the advantages of both RGB image and HSI, it is not only convenient to detect disease accurately but also affordable. However, recovering HSIs from RGB images is an ill-posed problem since a large amount of spectral information is lost when RGB sensors capture the light (Xiong et al. (2017)). Typically, the methods can be categorized into two types. The first one is to build relatively shallow learning models or sparse coding from a hyperspectral prior (Robles-Kelly (2015); Arad and Ben-Shahar (2016); Aeschbacher et al. (2017); Jia et al. (2017); Akhtar and Mian (2018)). Nonetheless, these methods have poor expression capacity and therefore have limited performance. Due to the high correlation between RGB values and corresponding hyperspectral radiance, the second category of methods is to learn a map between HSIs and RGB images by utilizing large amount of training data (Stiebel et al. (2018); Wang and Wang (2021)). Recently, deep learning methods have been introduced into spectral recovery tasks and have good performance (Shi et al. (2018); Zhao et al. (2020); Zhu et al. (2021)). Based on U-Net, Yan et al. (2018) proposed a multi-scale CNN called SRMSCNN, the encoder and decoder of the network are symmetrical and the symmetrical downsampling-upsampling architecture jointly encode image information for spectral reconstruction. Can and Timofte (2018) proposed a model called SREfficientNet which contains multiple residual blocks to utilize low-level features, through combing local residuals with global residuals to enhance the feature expression ability, this method requires much less computing resources to complete the reconstruction task.

This study is performed aiming to explore an effective and cost-savings way in disease detection application, and the spectral recovery disease detection model is proposed. The main contributions of this study arise from two aspects. First, the novel spectral recovery disease detection framework which has provided a new way of thinking for plant disease detection is proposed. Second, the maize spectral recovery dataset is built and the effect of spectral recovery model on recovery performance is explored. By using the framework we proposed, the recovered maize HSIs are reconstructed from RGB images and the recovered HSIs perform well in disease detection, especially in complex environment scenarios. This means that we can use RGBimages to achieve nearly the same disease detection accuracy compared with HSIs.

## 2 Materials and methods

### 2.1 Dataset preparation

#### 2.1.1 Data acquisition and calibration

**Figure 1:**
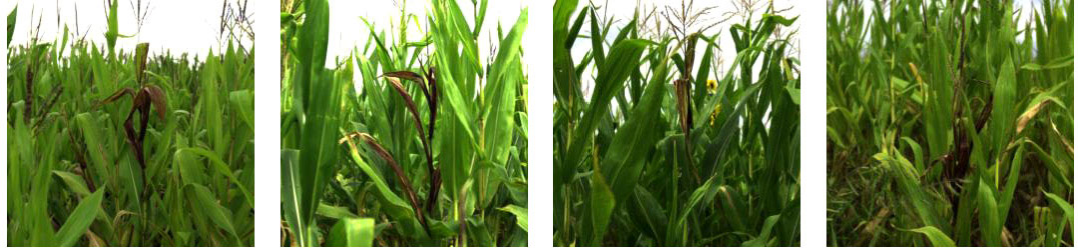
Part of maize samples in dataset.


Maize plants are cultivated in field, which is located in the Agricultural Experimental Base of Jilin University, Changchun, Jilin Province, China (125°25’43” E, 43°95’18” N). The variety of maize is Xianyu 335. To facilitate the speed and accuracy of spectral recovery from pest-infected maize RGB images, we obtained plenty of HSIs and corresponding RGB images of pest-infected maize leaves during mid-August. Each image data we collected contains both healthy and diseased maizes. Part of samples in dataset are shown in **Figure 1**. During the process of data collection, the data we obtained may suffer distortion due to the influence of intensity of illumination. It is essential to calibrate raw hyperspectral image by using white and dark references, according to Eq. 1. We carried a neutral reference panel and calibrated when is necessary so that the reliability of data is guaranteed.

(1) $$\begin{array}{l} \underline{{\text{I}}_{c}}=\frac{\underline{{\text{I}}_{r}}-\underline{{\text{I}}_{d}}}{\underline{{\text{I}}_{w}}-\underline{{\text{I}}_{d}}}, \end{array}$$

where, $\underline{{\text{I}}_{c}}$ and $\underline{{\text{I}}_{r}}$ refer to calibrated and raw hypersepctral images respectively, $\underline{{\text{I}}_{w}}$ and $\underline{{\text{I}}_{d}}$ refer to white and dark image respectively.

The hyperspectral sensor used for collecting data was the Specim IQ sensor (Specim, Oulu, Finland), which is an integrated system that could obtain and visualize HSIs and RGB images data. The Specim IQ camera provides 512×512 pixels images with 204 bands in the 400-1000 nm range. The RGB images and raw HSIs were captured by the Specim IQ simultaneously to avoid pixel position deviation. The integration time was automatically calculated by camera due to the light condition was unfixed. Owing to our goal is to recovery HSIs from natural RGB images and the wavelength of natural RGB images ranges from about 400 - 700 nm. For the purpose of reducing training cost and improving training efficiency, the images were resampled to 31 spectral bands in the visual range from 400 nm to 700 nm with a spectral resolution of 10 nm (Arad et al. (2022)). In this study, the images of maize were captured at a distance of 1-1.5 m. A neutral reference panel with 99% reflection efficiency was used to perform spectral calibration.

#### 2.1.2 Data preprocessing and augmentation

In order to relieve the burden of network and increase training samples, the hyperspectral data $\underline{X}\in {\mathbb{R}}^{C\times H\times W}$ and corresponding RGB data were divided into bunches of 31×128×128 and 31×128×128 patches respectively. The number of patches generated by an image depends on the stride, according to Eq. 2. To improve the generalization ability of the model, rotation and flipping were adopted to augment the original data.


(2) $$\begin{array}{l} N_{p}={\left(\frac{W-W_{p}}{S}+1\right)}^{2}, \end{array}$$

where, *N_p_* refers to the number of patches, *S* refers to stride, *W* and *W_p_* refer to the width of image and patch, respectively.

### 2.2 Spectral recovery and disease detection framework

**Figure 2:**
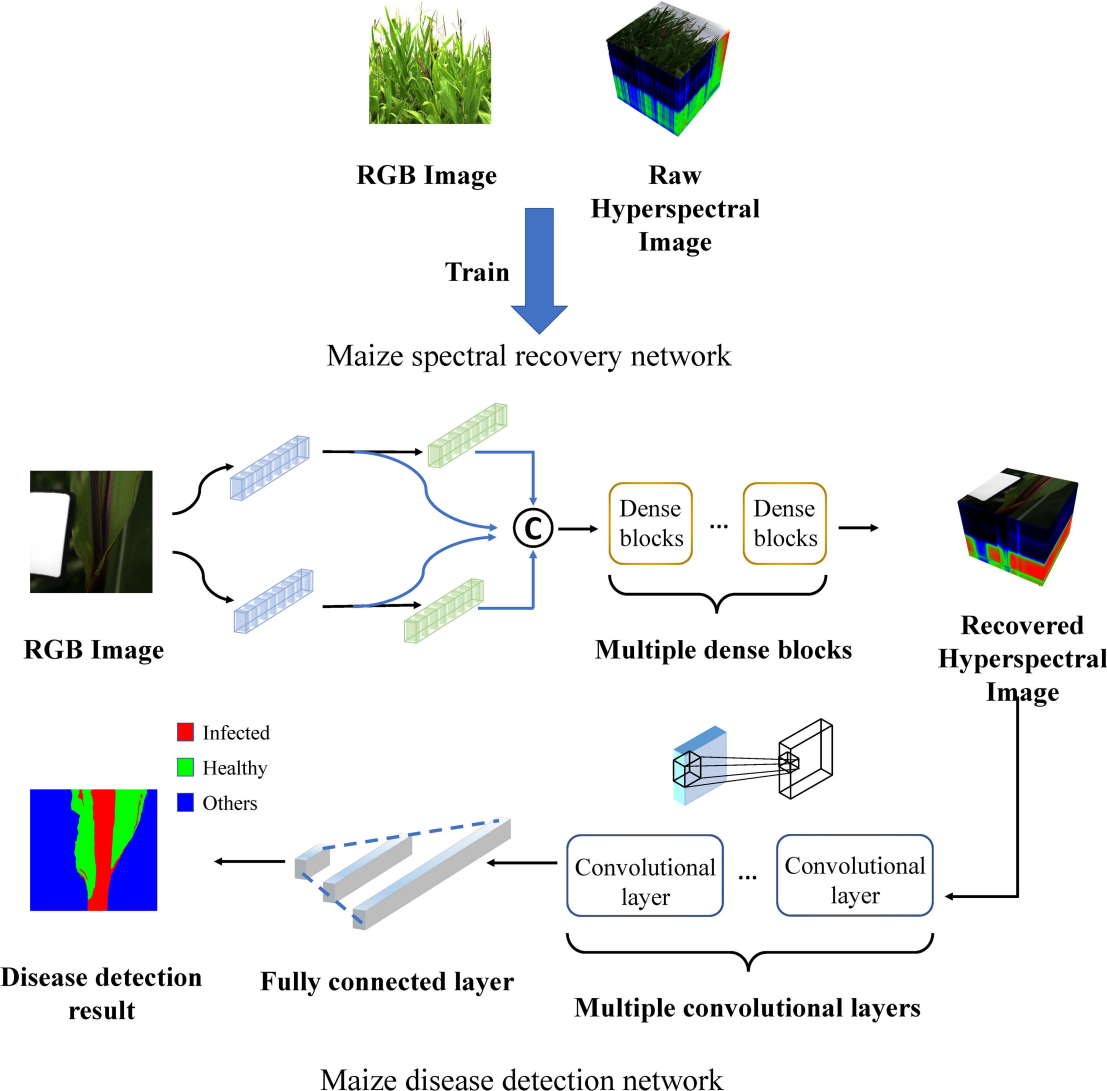
Schematic diagram of the overall maize spectral recovery and disease detection network architecture.

The overall framework is as depicted in **Figure 2**. The maize spectral recovery neural network was first trained by RGB images and corresponding raw HSIs. Raw RGB images were fed into the maize spectral recovery neural network, through feature extraction, mapping and reconstruction, we got the reconstructed HSIs. Subsequently, we put the reconstructed HSIs into disease detection neural network as input, and finally completed disease detection task. The detailed structure is described in the subsequent sections.

#### 2.2.1 Maize spectral recovery neural network

Recovering hyperspectral images from RGB images is an ill-posed problem, since a large amount of information is lost during the process of integrating the hyperspectral bands into RGB values. Traditional spectral recovery methods need hand-crafted priors (Arad and Ben-Shahar (2016); Akhtar and Mian (2018)), which performance is barely satisfactory due to the lacking of representing capacity. However, deep learning method, which performs well in many computer vision tasks, has been applied to hyperspectral recovery successfully. Through feeding a large number of training data, deep neural network can learn a map between RGB and HSIs. Various network structures have been proposed to accomplish the spectral recovery tasks, such as CNN and Generative Adversarial Network (GAN) (Zhang et al. (2022)). The GAN model contains a generator and a discriminator. The generator learns to reconstruct HSIs from RGB images and the discriminator judges whether the reconstruction quality is satisfactory. Although GAN can recover HSIs well, training GAN is unstable and likely to arise mode collapse. We tend to choose a more stable model. Recently, deep CNN based methods have achieved promising performance (Koundinya et al. (2018); Li et al. (2020); Fu et al. (2020)). In the training process of deep neural networks, the problem of the vanishing of the gradient may arise at times. The residual structure and dense structure could solve this problem. The residual structure could add skip connections among layers and provides the possibility for deeper network. However, the residual structure directly adds parameters of all previous layers which could destroy the distribution of convolution output and thus could reduce the transmission of feature information. In our maize spectral recovery network, we aim to make better use of spectral characteristics and thus the dense structure which concatenates channel dimensions of previous layers was adopted. The advanced hyperspectral recovery convolutional neural network (HSCNN+) contains dense blocks and could learn abundant and natural spectral information. In addition, unlike hyperspectral recovery convolutional neural network (HSCNN) requires prior knowledge from the RGB camera hardware, HSCNN+ requires no pre-knowledge from the RGB sensor and makes our framework easier to apply to field robots for agriculture. Therefore, the HSCNN+ which has superior performance on spectral recovery tasks was adopted as the backbone of our maize spectral recovery neural network (MSRNN).

**Figure 3:**
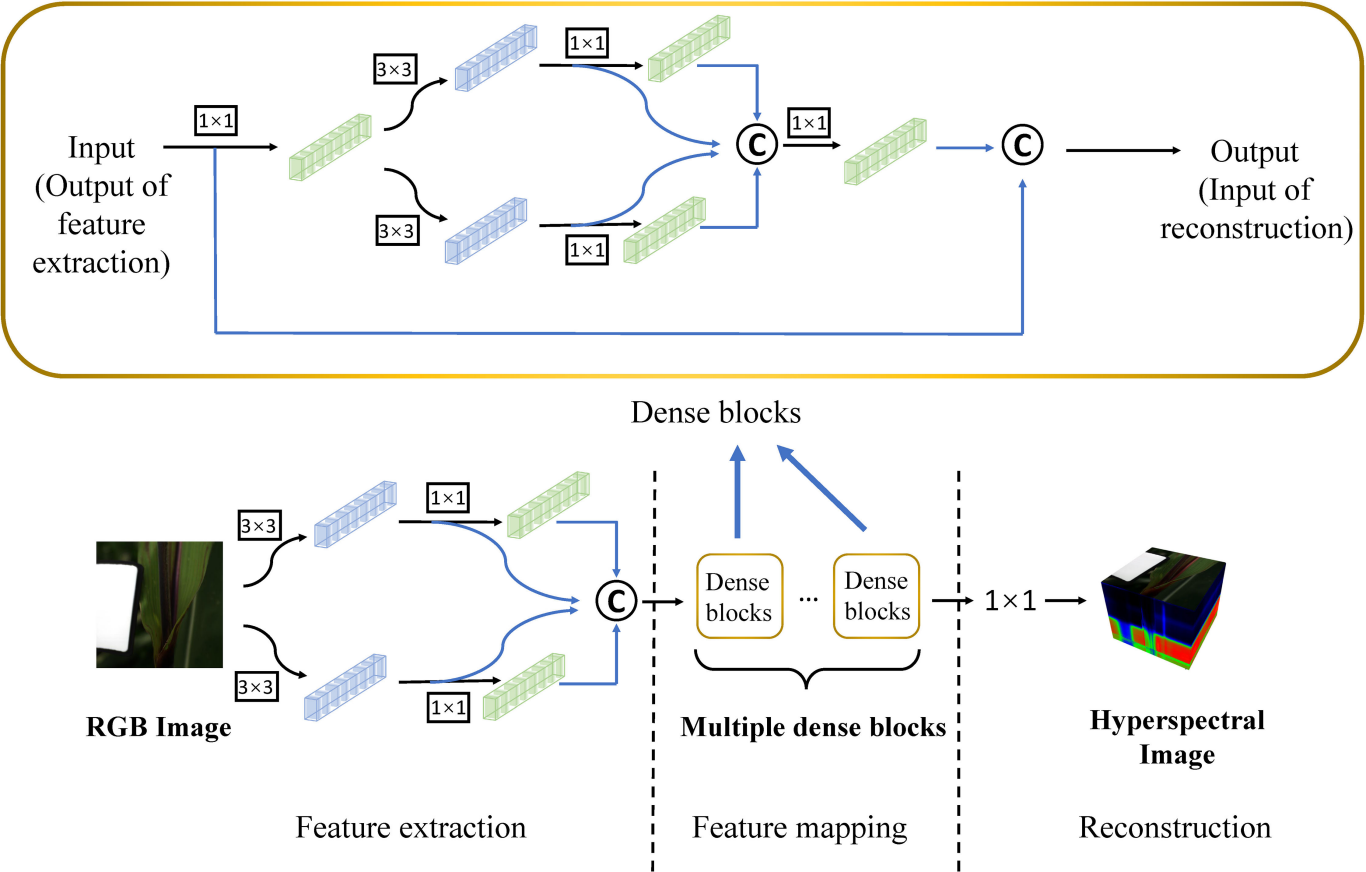
Network structure of the HSCNN+. The notation “1 × 1” and “3 × 3” denote the convolution with the kernel size of 1 × 1 and 3 × 3 respectively. The notation with rectangular box denotes the convolution is followed by ReLU activation function. The notation “C” with a circular box denotes the concatenation operation.

The HSCNN is one of the first CNN-based spectral recovery network and the HSCNN+ network was optimized on the basis of HSCNN (Xiong et al. (2017)).The HSCNN+ network include three parts which consists of feature extraction, feature mapping and reconstruction. The network structure is depicted in **Figure 3**. The core part of the network is the feature mapping part which contains multiple dense blocks. The output of previous layer mapped by 1 × 1, 3 × 3 and 3 × 3 - 1 × 1 convolution and then concatenated together. The dense structure enables the *l^th^* layer to receive the features from all preceding layers which can efficiently alleviate the problem of gradient vanishing, and what’s more, it offers a probability for deeper neural network. Our MSRNN has three parts, among them the structure of the first part of feature extraction and the last part of reconstruction is identical to the HSCNN+. The feature mapping part contains 20 dense blocks.

#### 2.2.2 Maize disease detection neural network

In terms of plant disease detection, most people focus on image-wise plant disease detection. However, it seems impossible for image-wise maize disease detection network to apply in field due to the influence of planting density. For pixel-wise plant disease detection, a large amount of spectral data is required. Fortunately, HSI is a good choice, and therefore CNN for HSIs classification was adopted as our pixel-wise maize disease detection neural network. The high dimensional data is sent into convolutional layers as input, and the output of convolutional layer is sent into a classifier which contains fully connected layer. All pixels in the spatial domain of hyperspectral images are classified into three classes: pest-infected maize, healthy maize, and others.

#### 2.2.3 Details of model training

For spectral recovery network, the dataset we used contains 100 maize HSIs, and the training set: test set is 9: 1. We set S in Eq. 2 to 16, so each HSIs may create 625 augmented patches for training. We used the Adam solver for optimization and beta set as 0.9. The learning rate is decayed with a cosine annealing from 0.001 to 0.00001, and we stop training when no obvious decay of training loss is observed. The loss function we used is MSEloss that measures the mean squared error (squared L2 norm) between each element in the input and target.

For disease detection network, the data we used is the output of spectral recovery network. For input HSIs, we created patches with stride of 2, and the training set: test set is 9: 1. The learning rate was set to 0.001 and the cross entropy function was used as the loss function.

### 2.3 Evaluation metrics

For the purpose of evaluating the quality of spectral reconstruction, Mean Relative Absolute Error (MRAE) and Root Mean Square Error (RMSE) were selected as evaluation metrics. MRAE computes mean absolute value between all spectral bands of recovered spectral images and groundtruth images. It represents the quality of spectral recovery and it is defined as Eq. 3. RMSE computes the root mean square error between the recovered and groundtruth spectral images. It is defined as Eq. 4.


(3) $$\begin{array}{l} MRAE=\frac{1}{N}\sum_{i=1}^{N}\frac{\left|I_{R}^{i}-I_{G}^{i}\right|}{I_{G}^{i}}, \end{array}$$

(4) $$\begin{array}{l} RMSE=\sqrt{\frac{1}{N}\sum_{i=1}^{N}{\left(I_{R}^{i}-I_{G}^{i}\right)}^{2}}, \end{array}$$

where, *N* refers to the total number of pixels, $I_{R}^{i}$ and $I_{G}^{i}$ refer to the *i^th^* pixel of the recovered spectral images and groundtruth images respectively.

In addition to verifying the quality of the spectral recovery model through the above evaluation metrics, we utilize a pest-infected maize detection model to test the effectiveness of the spectral recovery model. This model classifies pixel-wise images into three classes: infected part, healthy part and others. The class “others” means it neither belongs to healthy maize nor infected maize, such as hand, white panel, stones and so on. When the agriculture robots are working in field, they may snap to something that does not relate to maize and could disturb the detection results. These things are therefore classified to “other”. We chose precision, recall and F1 score to evaluate our disease detection model. These evaluation metrics can be calculated by Eqs 5, 6, 7. We also used the overall accuracy (OA) and average accuracy (AA) evaluation metrics to evaluate the detection ability of the model. Here, OA refers to the total number of correctly classified pixels divided by the total number of all pixels and AA refers to the sum of accuracy for each class predicted divided by the number of class.


(5) $$\begin{array}{l} P=\frac{T_{P}}{T_{P}+F_{P}}, \end{array}$$

(6) $$\begin{array}{l} R=\frac{T_{P}}{T_{P}+F_{N}}, \end{array}$$

(7) $$\begin{array}{l} F_{1}=\frac{2P\times R}{P+R}, \end{array}$$

where, *P* refers to precision, *R* refers to recall, *F*_1_ refers to F1 score, *T_P_* refers to the number of true positives, *F_P_* refers to the number of false positives, and *F_N_* refers to the number of false negatives.

## 3 Experiments and discussion

All the image preprocessing processes and main algorithm were conducted using MATLAB R2021a, Anaconda3 (Python 3.8), PyTorch library, scikit-learn library, etc. The proposed model was trained and tested with hardware configuration including IntelR i9-10980XE CPU (3.00GHz), 64-GB memory, and NVIDIA RTX A5000 (CUDA 11.4) graphics card.

### 3.1 Evaluation of spectral recovery quality

For maize RGB images to HSIs conversion, the HSCNN+ which we chose for maize spectral recovery was compared with several state-of-the-art algorithms (Zamir et al. (2020); Cai et al. (2022); Zhao et al. (2020); Shi et al. (2018)). The dataset we used was mentioned in section 2.1, and the test set was strictly never used for training. All compared models adopted same patch size as HSCNN+. The initial learning rate of HRNet was 1×10^-4^. For MST++ and MIRNet, the learning rate was set to 4×10^-4^ and halved every 50 epochs during the training process. The batch size was 20. Random flipping and rotation were used for data augmentation. In order to evaluate the effectiveness of HSCNN+, we used MRAE and RMSE evaluation metrics. The experimental results are shown in **Table 1**.

**Table 1:** MRAE and RMSE results of RGB to hyperspectral conversion.

Model	MRAE	RMSE
MST++	0.1681	0.1220
MIRNet	0.3073	0.1310
HRNet	0.1120	0.1289
**HSCNN+**	**0.0713**	**0.1204**

The bold values mean the MRAE and RMSE of HSCNN+ is the lowest among four models.

**Table 1** gives the numerical results of different models on the test set. As can be seen, the MRAE of HSCNN+ reached 0.0713 which was lower than MST++ 0.1681, MIRNet 0.3073, HRNet 0.1120. The HSCNN+ model achieved 57.6%, 76.8%, 36.3% decrease in MRAE compared with MST++, MIRNet, HRNet respectively. The RMSE of HSCNN+ were lower than all compared models as well and achieved 1.3%, 8.1%, 6.6% reduction. It demonstrates that in the maize spectral recovery case, the model learned by HSCNN+ is more suitable and can be well generalized. fidelity of the HSCNN+ model in maize spectral recovery application. However, it can be observed that the 228 largest error happens at both ends of the spectral bands. To the best of our knowledge, this may be caused 229 by the acquisition accuracy difference of the spectral camera. The precision of camera in middle bands is 230 higher than ends of the spectral bands. Therefore, the error at both ends of spectral bands caused by data 231 collection may impact on training accuracy. Fortunately, both ends of spectral bands have little impact on the overall disease detection accuracy.

**Figure 4:**
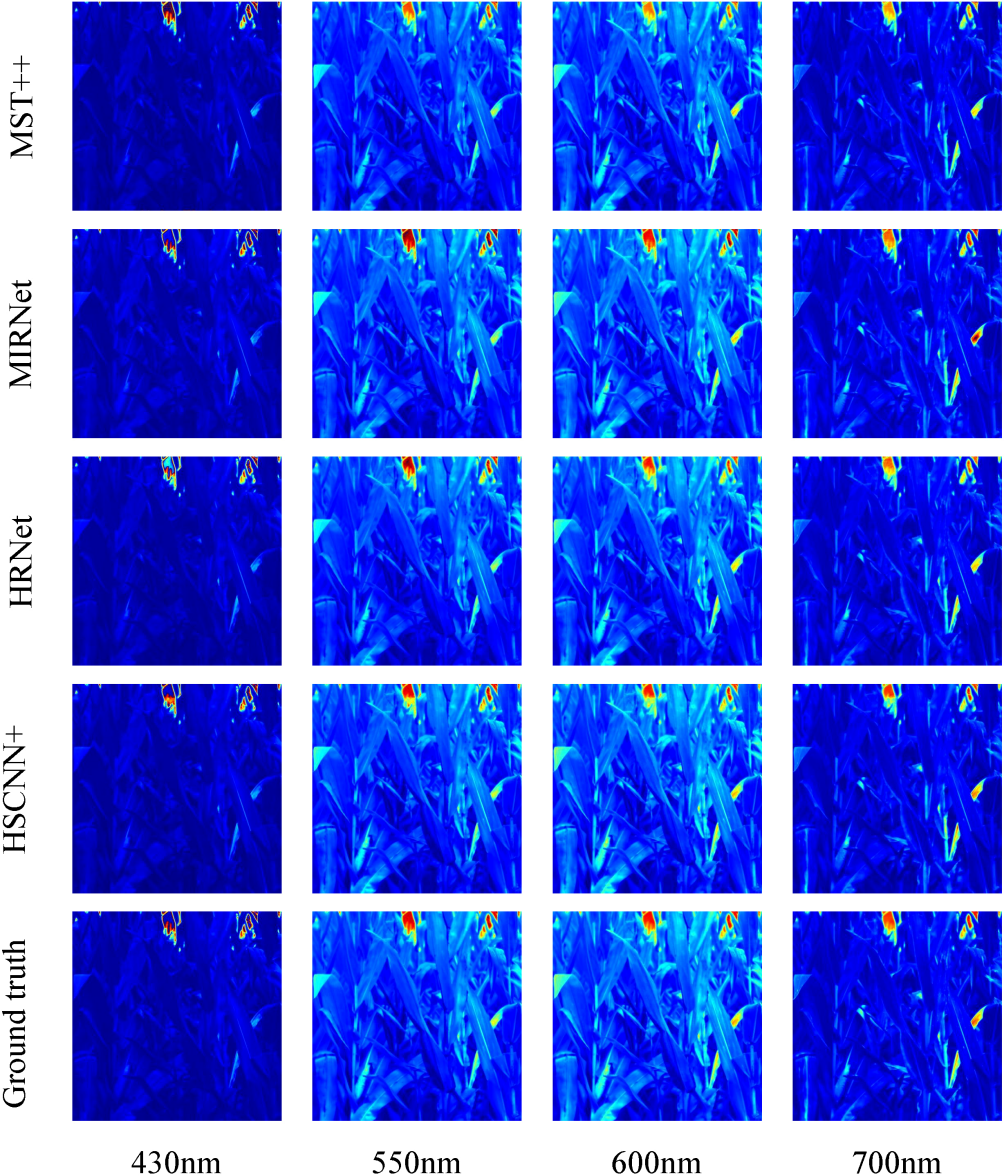
Visual comparison of four selected bands for maize spectral recovery from RGB images.

**Figure 5:**
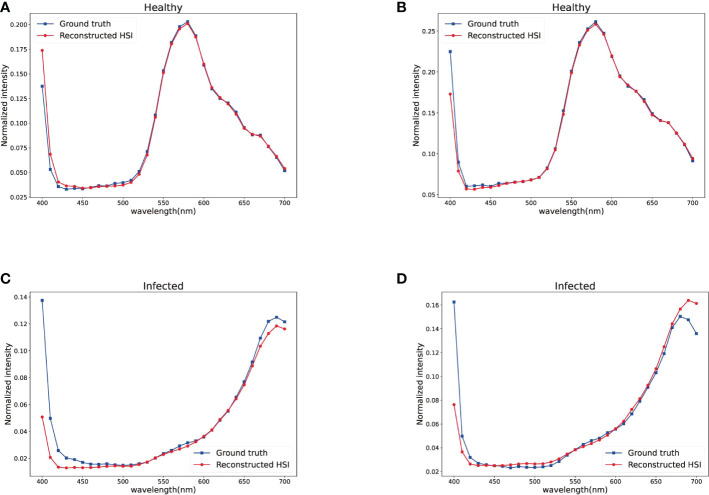
Signature of four selected spatial points in **Figure 4**. **(A)** Point (133,81) of healthy part. **(B)** Point (307,439) of healthy part. **(C)** Point (304,191) of infected part. **(D)** Point (353,277) of infected part.

To evaluate the perceptual quality of maize spectral reconstruction, **Figure 4** shows the visual results of four selected bands from a test hyperspectral image. The first four rows show the data distribution of 5 methods and the ground truth in the last row. As shown in **Figure 4**, the spectral recovery model maintained the spatial features well and the HSCNN+ model kept more spectral details than other compared models. As a result of most of the recovered HSIs are maize leaves which have similar spectral characteristics, details information in dark parts are not obvious, we recommend readers to concentrate on texture details. **Figure 5** further shows the spectral signatures of four selected points from the test data, two of them were selected randomly from healthy part and two others were selected randomly from infected part. The recovered HSI and ground truth HSI have 31 spectral bands from 400 nm to 700 nm. We can observe that the spectral curve of reconstructed HSI has high similarity with ground truth, which confirmed the high reconstruction fidelity of the HSCNN+ model in maize spectral recovery application. However, it can be observed that the largest error happens at both ends of the spectral bands. To the best of our knowledge, this may be caused by the acquisition accuracy difference of the spectral camera. The precision of camera in middle bands is higher than ends of the spectral bands. Therefore, the error at both ends of spectral bands caused by data collection may impact on training accuracy. Fortunately, both ends of spectral bands have little impact on the overall disease detection accuracy.

### 3.2 Comparison of disease detection network in different scenarios

**Figure 6:**
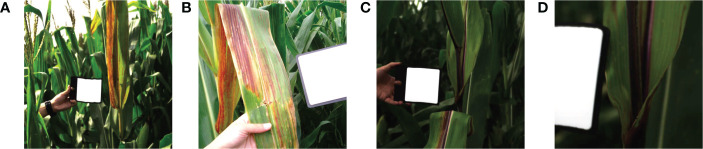
Four scenarios. **(A)** Scenario 1. **(B)** Scenario 2. **(C)** Scenario 3. **(D)** Scenario 4.

According to the above experiment results, we found that HSCNN+ is more suitable for maize spectral recovery. Raw maize RGB images was converted to reconstructed HSIs by maize spectral recovery net. In order to test the effectiveness of our reconstructed HSIs in disease detection, we test the detection performance of recovered HSIs in different detection scenarios. The maize spectral recovery disease detection framework is intended to apply in field robots for disease detection. Therefore, it is essential to choose scenarios that field robots are likely to be encountered. The four scenarios include three close shot and one complex scene. When the agriculture robots are working in field and moving between plants, the scenarios we chose for test are likely to be appeared in the robot view. We used our disease detection model and the input of models were raw RGB images, reconstructed HSIs and raw HSIs, so that we could clearly see the performance of reconstructed HSIs. The HSI and RGB image data collected in field were chosen as test detection scenarios as shown in **Figure 6**. The raw data of these four scenarios has never been used for our maize spectral recovery. We fed in the raw RGB images of different scenarios into maize spectral recovery network to get recovered maize HSIs, then the reconstructed HSIs, raw RGB images and raw HSIs were imported into maize disease detection network to finally get the disease detection results.

**Figure 7:**
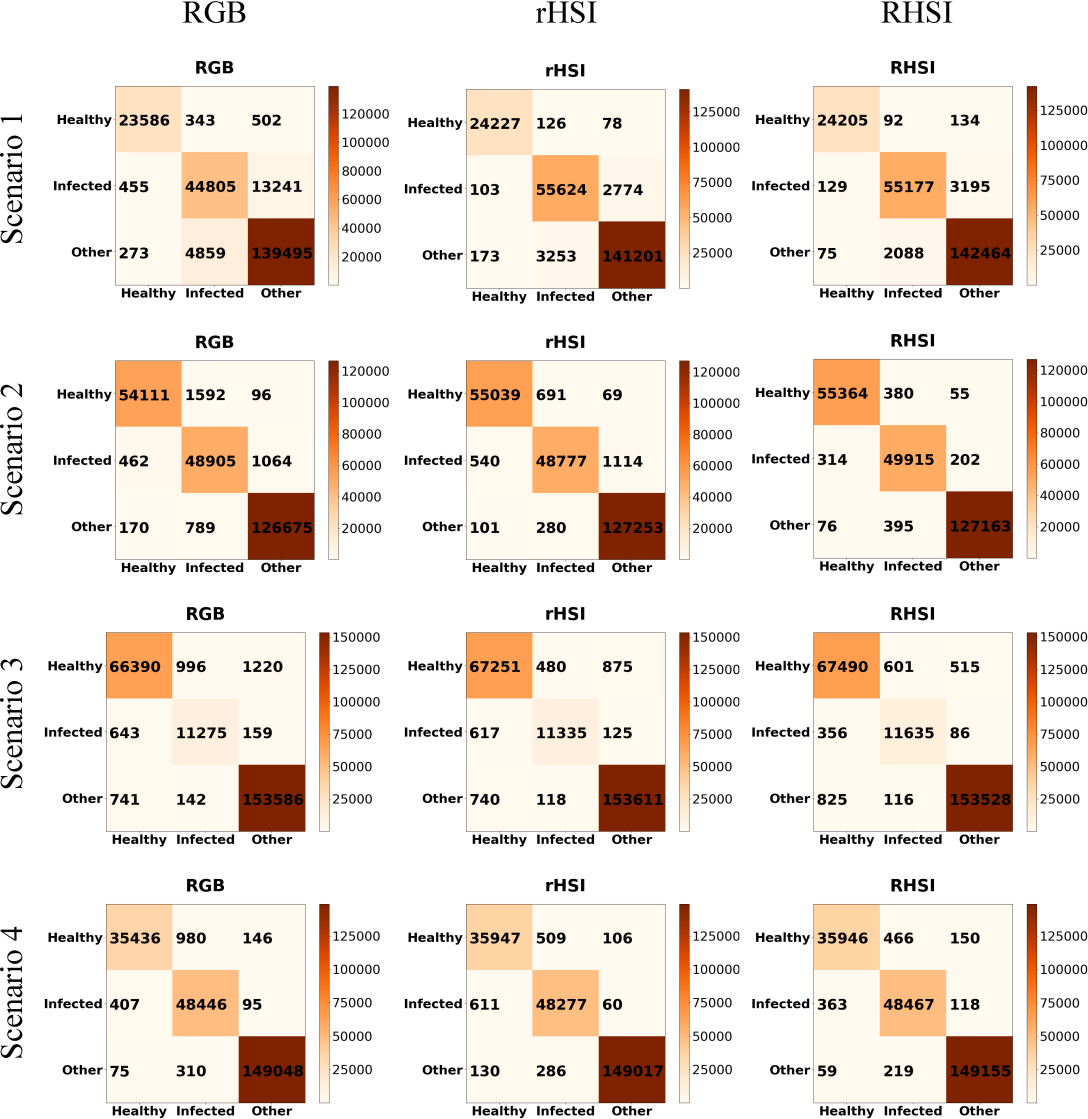
Confusion matrices of all scenarios. (In each confusion matrix, the abscissa axis represents predicted class and the ordinate axis represents actual class.).

Our maize disease detection network concentrated on pixel-wise detection, all pixels of HSIs were used as dataset and the HSIs size is 512×512. The disease detection model contains 3D and 2D convolutional layers to extract features in spectral and spatial domain, and end up with fully connected layers as classifier to classify pixels into three classes: healthy, infected and others. The total number of labeled pixels in scenario1, scenario2, scenario3 and scenario4 are 227559, 233864, 235152 and234614 respectively. The 253 experiment results are shown in **Table 2**, and **Figure 7** gives a detailed account of the disease detection results 254 in all scenarios.

**Table 2:** Detection results of maize disease in different scenarios.

	Scenario 1 (complex)			Scenario 2			Scenario 3			Scenario 4		
	RGB	RHSI	rHSI	RGB	RHSI	rHSI	RGB	RHSI	rHSI	RGB	RHSI	rHSI
Precision(%)												
Healthy	97.01	**99.16**	98.87	98.85	**99.3**	98.85	97.96	**98.28**	98.02	98.66	**98.84**	97.98
Infected	89.6	**96.2**	94.27	95.36	**98.47**	98.05	90.83	94.2	**94.99**	97.41	**98.61**	98.38
Others	91.03	97.72	**98.02**	99.09	**99.8**	98.35	99.11	**99.61**	99.35	99.84	99.82	**99.89**
Recall(%)												
Healthy	96.54	99.07	**99.16**	96.97	**99.22**	98.64	96.77	**98.37**	98.02	96.92	**98.32**	**98.32**
Infected	76.59	94.32	**95.08**	96.97	**98.98**	96.72	93.36	**96.34**	93.86	98.97	**99.02**	98.63
Others	96.45	**98.5**	97.63	99.25	99.63	**99.7**	99.43	99.39	**99.44**	99.74	**99.81**	99.72
F1(%)												
Healthy	96.77	**99.12**	99.02	97.9	**99.26**	98.74	97.36	**98.33**	98.02	97.78	**98.58**	98.15
Infected	82.58	**95.25**	94.68	96.16	**98.72**	97.38	92.08	**95.26**	94.42	98.18	**98.81**	98.5
Others	93.66	**98.11**	97.83	99.17	**99.71**	99.39	99.27	**99.5**	99.4	99.79	**99.82**	99.81
**OA(%)**	91.35	**97.49**	97.14	98.22	**99.39**	98.8	98.34	**98.94**	98.74	99.14	**99.41**	99.28
**AA(%)**	89.86	**97.3**	97.29	97.73	**99.28**	98.35	96.52	**98.03**	97.11	98.55	**99.05**	98.89

a RGB means RGB image, RHSI means raw hyperspectral image, rHSI means recovered hyperspectral image.The bold values mean the best result among RGB, RHSI and rHSI in different scenarios

**Table 2** compares the performance of different data in four test scenarios. As can be seen, the OA of disease detection reached RGB 91.35%, RHSI 97.49%, rHSI 97.29% in scenario 1, reached RGB 98.22%, RHSI 99.39%, rHSI 98.80% in scenario 2, reached RGB 98.34%, RHSI 98.94%, rHSI 98.74% in scenario 3, and reached RGB 99.14%, RHSI 99.41%, rHSI 99.28% in scenario 4. We found that in all scenarios, the OA of disease detection using reconstructed HSIs were all higher than that using RGB images which means our reconstructed HSIs performed better than RGB images. Moreover, although the OA of detection when using reconstructed HSIs were slightly lower than that when taking raw HSIs as input, the detection performance between using raw HSIs and recovered HSIs were very close. In most cases, not only the OA metrics, almost all evaluation metrics including precision, recall, F1 score and AA follow the above rules. This means that our reconstructed HSIs would work just as well as raw HSIs and better than raw RGB images. Above all, our recovered HIS has been achieved relatively large improvement in detecting infected maize compared with raw RGB image. In some cases, RGB image itself already has a high accuracy, the major reason for this is that in a relatively simple scenario, there is less disturbance. Therefore, the information raw RGB images provided match with the corresponding algorithms could achieve relatively high accuracy. It is difficult for our recovered HSIs to achieve great improvement and the space for improving is seriously limited.

To validate the proposed model’s detection results, we performed a 5-fold cross-validation strategy. **Table 3** summarizes the disease detection OA in different test scenarios of all 5-folds. It could be observed that the recovered HSIs performed well to improve the detection accuracy in all folds which indicates the generalization capabilities of the framework.

**Table 3:** Detection OA (%) of individual folds in the 5-fold cross validation process.

	Scenario 1 (complex)			Scenario 2			Scenario 3			Scenario 4		
Fold no.	RGB	RHSI	rHSI	RGB	RHSI	rHSI	RGB	RHSI	rHSI	RGB	RHSI	rHSI
Fold 1	92.69	97.06	**97.42**	97.51	**99.55**	97.70	97.98	98.75	**99.09**	99.35	**99.61**	99.22
Fold 2	94.56	**97.19**	97.08	98.13	**99.45**	99.39	98.34	99.07	**99.19**	99.13	99.58	**99.60**
Fold 3	90.87	**97.38**	97.18	96.05	99.34	**99.41**	98.63	**99.16**	98.78	99.28	**99.54**	99.13
Fold 4	93.55	**97.49**	97.30	97.60	**99.63**	99.22	98.71	**99.15**	99.09	98.57	99.43	**99.56**
Fold 5	93.51	**97.41**	97.19	99.13	**99.58**	99.30	98.07	**99.17**	99.16	99.39	**99.59**	99.54
Average	93.04	**97.31**	97.23	97.68	**99.51**	99.00	98.35	**99.06**	**99.06**	99.14	**99.55**	99.41

a RGB means RGB image, RHSI means raw hyperspectral image, rHSI means recovered hyperspectral image.The bold values mean the best result among RGB, RHSI and rHSI in different scenarios.

**Figure 8:**
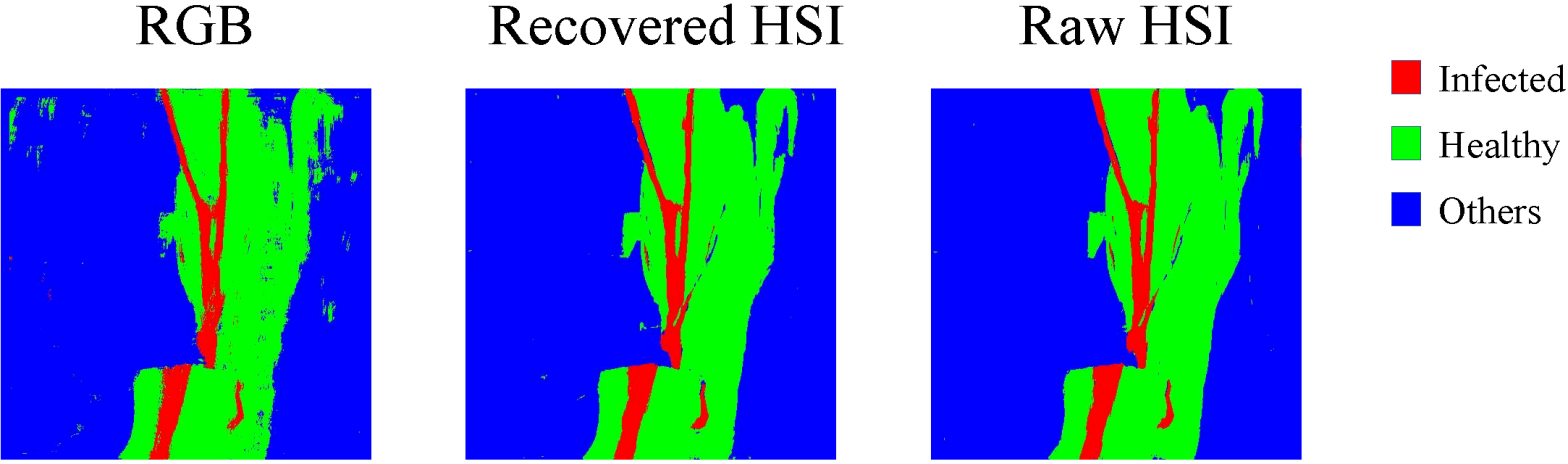
Visual comparison of disease detection using RGB image, recovered HSI. and raw HSI.

**Figure 7** shows the confusion matrices of all scenarios. The abscissa axis and ordinate axis of each confusion matrix represents predicted class and actual class respectively. As can be seen, the great mass of pixel samples distribute on the diagonal line of confusion matrices. In most cases, the diagonal numbers in rHSI are greater than in RGB, which indicates that our reconstructed HSI as input data could support the detection model has higher accuracy than RGB image. For further test the effect of reconstructed HSI, we chose a scenario to visualize our detection results as shown in **Figure 8**. As depicted in **Figure 8**, using the recovered HSI to detect disease has higher stability and precision compared with using the RGB data.

From detection results in scenario 1, we observed that using the reconstructed HSIs has tremendous effects on performance of disease detection. By importing raw RGB data into spectral recovered network to get recovered HSIs, the OA of disease detection is improved from 89.86% (using raw RGB images) to 97.29% (using recovered HSIs). This would be caused by the complex detection environment as shown in **Figure 6A**. The spatial features extracted by disease detection network from raw RGB images can not sufficient to support the disease detection tasks. By using spectral recovered network to convert raw RGB images to recovered HSIs, the spectral features were enlarged. Compared with 3 spectral channels in RGB images, the reconstructed HSIs have 31 channels which could get more accurate disease detection in the complex scenes.

Above all, the maize spectral recovery network first trained by our maize spectral recovery dataset which contains maize RGB images and corresponding HSIs to learn a map between raw RGB data and HSIs data. After enhancing spectral features of raw RGB images, the recovered HSIs can perform as well as raw HSIs in disease detection application. This means that we could obtain original maize RGB data fast by a low-cost digital camera, and then throw into our maize spectral recovery network to get reconstructed maize HSIs. By utilizing the recovered maize HSIs to detect diseases, we could achieve almost the same accuracy as raw HSIs can do. In view of the high-cost and time-consuming of acquiring HSIs and the operational complexity of hyperspectral camera, we offer a better choice for field maize disease detection application.

## 4 Conclusion

This research proposed a maize spectral recovery disease detection framework based on HSCNN+ and maize disease detection CNN to complete low-cost and high-precision maize disease detection in field application. We found ideal spectral recovered model to reconstruct HSI data from raw maize RGB data and used the recovered HSI data as input for disease detection network. The spectral information in the raw data was expanded, and the quality of HSI reconstruction was satisfactory. Our framework effectively improved the disease recognition accuracy when taking RGB images as raw data and had achieved excellent results in disease detection. The experiment findings demonstrated the efficiency and practicability of our framework, and it is successfully accomplished to detect infected maize under various conditions especially in the complex environment conditions. In the future, we plan to combine our theory with practice to resolve problems in agriculture production. The disease detection agricultural robots need to receive real-time data to make quick judgement. On account of the high-cost and time-consuming characteristics of the hyperspectral imaging system, it is almost impossible to apply it to field real-time disease detection. However, the framework we proposed offers this possibility.

## Data availability statement

The raw data supporting the conclusions of this article will be made available by the authors, without undue reservation.

## Author contributions

JL and RZ prepared materials and used the hyperspectral camera to obtain hyperspectral images. JF, JL, and RZ wrote the manuscript. JF and RZ provided funding for this work. ZC made guidance for the writing of the manuscript. JL, RZ, and YQ designed the experiment. DL provided guidance for revising manuscript. All authors contributed to the article and approved the submitted version.
